# Supplementary material for: From sequence to enzyme mechanism using multi-label machine learning
Source: BMC Bioinformatics. 2014 May 19;15:150. doi: 10.1186/1471-2105-15-150 (PMC4229970; doi:10.1186/1471-2105-15-150)
Supplement: Additional file 2 — Java code of ml2db. Additional file ml2db_code.tar.gz contains the Java source code to run the multi-label machine learning experiments and save the results to database. The code’s Javadoc is included. [file 1471-2105-15-150-S2.zip › additional file 2/ml2db/utils/doc/uk/ac/standrews/utils/main/stats/PseudoTruncatedParetoSingleton.html]

PseudoTruncatedParetoSingleton


JavaScript is disabled on your browser.


- Overview
- Package
- Class
- Use
- Tree
- Deprecated
- Index
- Help

- Prev Class
- Next Class

- Frames
- No Frames

- All Classes

- Summary:
- Nested |
- Field |
- Constr |
- Method

- Detail:
- Field |
- Constr |
- Method


uk.ac.standrews.utils.main.stats

## Class PseudoTruncatedParetoSingleton

- java.lang.Object
- - uk.ac.standrews.utils.main.stats.PseudoTruncatedParetoSingleton

- ---

    

  ```
  public class PseudoTruncatedParetoSingleton
  extends java.lang.Object
  ```

  Class

  Version:
  :   4 Aug 2008

  Author:
  :   Luna De Ferrari luna.deferrari-at-ed.ac.uk

- - ### Method Summary

    Methods

    | Modifier and Type | Method and Description |
    | --- | --- |
    | `static PseudoTruncatedPareto` | `getInstance(double scale, double shape, int offset, int truncationThreshold, int lengthOfPseudoRandomList)` |
    | `PseudoTruncatedPareto` | `getPseudoTruncatedPareto()` |

    - ### Methods inherited from class java.lang.Object

      `equals, getClass, hashCode, notify, notifyAll, toString, wait, wait, wait`

- - ### Method Detail


    - #### getInstance

      ```
      public static PseudoTruncatedPareto getInstance(double scale,
                                      double shape,
                                      int offset,
                                      int truncationThreshold,
                                      int lengthOfPseudoRandomList)
      ```


    - #### getPseudoTruncatedPareto

      ```
      public PseudoTruncatedPareto getPseudoTruncatedPareto()
      ```


- Overview
- Package
- Class
- Use
- Tree
- Deprecated
- Index
- Help

- Prev Class
- Next Class

- Frames
- No Frames

- All Classes

- Summary:
- Nested |
- Field |
- Constr |
- Method

- Detail:
- Field |
- Constr |
- Method
